# A novel hydrophobin encoded by *hgfII* from *Grifola frondosa* exhibiting excellent self-assembly ability

**DOI:** 10.3389/fmicb.2022.990231

**Published:** 2022-09-09

**Authors:** Jiuxia Yang, Lu Ge, Bo Song, Zhongqiang Ma, Xiaotian Yang, Bo Wang, Yixin Dai, Haijin Xu, Mingqiang Qiao

**Affiliations:** ^1^NHC Key Laboratory of Critical Care Medicine, Tianjin First Central Hospital, Tianjin, China; ^2^The Key Laboratory of Molecular Microbiology and Technology, Ministry of Education, College of Life Sciences, Nankai University, Tianjin, China; ^3^The Key Laboratory of Bioactive Materials, Ministry of Education, College of Life Sciences, Nankai University, Tianjin, China

**Keywords:** *Grifola frondosa*, mycelium, hydrophobin, self-assembly, surface hydrophobicity

## Abstract

Hydrophobins are small proteins from filamentous fungi, which have remarkable self-assembly properties of great potential, e.g., as drug carriers and as anti-bacterial agents, but different hydrophobins, with improved properties, are needed. HGFI (a hydrophobin from *Grifola frondosa*) is a class I hydrophobin, which can self-assemble into rodlet structures with a length range 100–150 nm. In this study, we identified a new hydrophobin gene (*hgfII*) from the mycelium of *G. frondosa* with a much higher transcriptional level than *hgfI*. Heterologous expression of *hgfII* was accomplished in the *Pichia pastoris*. X-ray photoelectron spectroscopy and water contact angle assay measurements revealed that HGFII can self-assemble into a protein film at the air–solid interface, with circular dichroism and thioflavin T fluorescence studies showing that this effect was accompanied by a decrease in α-helix content and an increase in β-sheet content. Using atomic force microscopy, it was shown that HGFII self-assembled into rodlet-like structures with a diameter of 15–30 nm, showing that it was a class I hydrophobin, with self-assembly behavior different from HGFI. The surface hydrophobicity of HGFII was stronger than that of HGFI, meanwhile, in emulsification trials, HGFII displayed better dispersive capacity to the soybean oil than HGFI, producing a more stable and durable emulsion.

## Introduction

Hydrophobins are a kind of amphiphilic protein, which exist in various groups of fungi (Cooper and Kennedy, 2010). It has been reported that hydrophobin has been found in more than 20 kinds of fungus, especially the filamentous fungi (Linder et al., 2005). The primary structure of this kind of protein generally contains 70–120 amino acids, so its molecular weight is usually low (≤20 kDa) (Wosten and de Vocht, 2000). Hydrophobins are remarkably surface-active and can spontaneously assemble into an amphiphilic protein film at the air–liquid or hydrophobic/hydrophilic interface (Wosten, 2001). The results from genomic analysis of different species of fungi reveal that hydrophobins generally present as a family with a small number of members, although there are also some fungal species with many members, such as *Coprinus cinereus*, which contains 33 hydrophobins (Sunde et al., 2008; Littlejohn et al., 2012).

The identity of the amino acid sequences between hydrophobins is relatively low, but all the hydrophobins share similarities with respect to their primary structure. Hydrophobins typically contain eight Cysteine (Cys) residues, among which the second and third Cys residues are consecutive, as are the sixth and seventh Cys residues (Linder, 2009). Hydrophobins can be divided into two groups, class I and class II, according to their hydropathy plot, the number of amino acid residues in the C3–C4 loop, and their solubility (Heitman et al., 2012; Berger and Sallada, 2019).

It has been reported that the class I hydrophobins can self-assemble into rodlet-like structures. This structure is relatively robust, in that it is highly insoluble in water, 60% ethanol or 2% hot sodium dodecyl sulphate (SDS) aqueous solution, and can be depolymerized only in strong acid such as trifluoroacetic acid (TFA) (Kwan et al., 2006; Berger and Sallada, 2019). In contrast, the stability of the protein layer formed by class II hydrophobins is relatively low and it cannot withstand washing with 2% hot SDS solution (Paananen et al., 2003; Hou et al., 2009).

In most cases, hydrophobins exist as a component of a fungal structure, such as a coating on the surface of a fruiting body to make it hydrophobic (Lugones et al., 1996; Ren et al., 2013). Some hydrophobins have been found to be a constituent of the mycelium, helping the mycelium break down the air–liquid barrier (Askolin et al., 2005; Longobardi et al., 2012), whereas hydrophobins can also endow conidia with a degree of hydrophobicity, which promotes the spread of the spores in the air (Aimanianda et al., 2009). Due to the outstanding surface activity of hydrophobins, increasing research is being conducted into practical applications of hydrophobins, including protein immobilization (Linder et al., 2002; Qin et al., 2007), protein expression and purification (Mustalahti et al., 2013; Zhang et al., 2017), food preservation (Khalesi et al., 2016), sustained drug release (Sarparanta et al., 2012), and emulsification of foaming agents (Bailey et al., 2002), among other fields.

*Grifola frondosa* (*G. frondosa*) is an edible mushroom with high nutritional value and biological activity (Chen et al., 2017). At present, only one hydrophobin, HGFI, has been reported from *G. frondosa* (Yu et al., 2008). HGFI belongs to the class I hydrophobins, and the mature protein sequence contains 83 amino acids. It can self-assemble into rodlet structures with remarkable surface activity. Recently, HGFI has been widely tested in fusion proteins (Zhao et al., 2015), in the development of antibacterial materials (Wang et al., 2017a), dispersion of multi-walled carbon nanotubes (Wang et al., 2010) and as a biological drug carrier (Zhao et al., 2016).

In order to find more hydrophobins in *G. frondosa*, to provide greater choice for further applications, we report in the current study on the identification of a new hydrophobin gene *hgfII* in *G. frondosa*, and demonstrate its heterologous expression in *Pichia pastoris* (*P. pastoris*). Gene expression patterns of *hgfII* reveal significant differences from those of *hgfI*. The recombinant protein HGFII could also assemble into rodlets, as with other class I hydrophobins, but it formed rodlet structures with a unique self-assembly mechanism. Moreover, differences between HGFI and HGFII in terms of surface hydrophobicity and emulsification effect toward soybean oil were identified. These results provide new insights on the assembly properties of class I hydrophobins in *G*. *frondosa*, allowing us to speculate on and develop new and innovative fields of application.

## Materials and methods

### Strains, plasmids, and culture conditions

The *G. frondosa* fungal strain used in this study was CICC-50075, which was available in our laboratory. It was grown at 28°C for 15 days in either potato dextrose broth (PDB) liquid medium (potato 200 g/l, glucose 20 g/l) or potato dextrose agar (PDA) (potato 200 g/l, glucose 20 g/l, and agar 15 g/l) solid medium. The *Escherichia coli* vector strain pPIC9 (carrying the *his*4 gene) and the *P. pastoris* strain GS115 (His4^−^ Mut^S^) were used for heterologous expression of the *hgfII* gene. The *E. coli* strains were cultivated in Luria broth (LB) (yeast extract 5 g/l, tryptone 10 g/l, NaCl 10 g/l) medium at 37°C for 16 h, while the yeast strain was cultivated in buffered minimal medium (BMM) (for shake flask fermentation) or BSM medium base (for fermentation) at 28°C for 96 h.

### RNA extraction and cDNA synthesis

The RNA Extraction Kit (RN5350G; Shanghai, China) was used to extract the total RNA from the fungal mycelium. The cDNA was synthesized with the cDNA Amplification Kit (M5621S; Shanghai, China). PCR reaction were performed using the Powerscript Reverse Transcriptase Kit using the Applied Biosystems thermal cycler (ABI2720; Shanghai, China) according to the manufacturer’s instructions.

### Identification and characterization of *hgfII*

In order to find new hydrophobin genes of *G. frondosa*, we first constructed a cDNA library of the mycelium as previously described (Yu et al., 2008). The cDNA was cloned into the *E. coli* pGADT vector and transformed into DH5α cells. PCR amplification and sequencing were used to screen positive transformants. The inserted fragments of 0.5–2.0 kb were selected for further verification. To identify whether it is a hydrophobin gene, we searched whether the corresponding amino acid sequence contains the conserved 8 Cys.

The translated protein sequence of hydrophobin-like gene was completed using the Sequencher program (version 4.5). Primers *hgfII* gF/gR (Supplementary Table S1) were used to amplify the full-length genomic DNA sequence of *hgfII*. The predicated signal peptide of the translated protein was identified using SignalIP 5.0 Server (Nielsen et al., 2019). Sequence alignment was carried out using DNAMAN software (version 6.0). The hydropathy plot analyses were accomplished using Hphob./Kyte & Doolittle scale.^1^

### The quantitative real-time PCR analysis

For the quantitative real-time PCR (qPCR) assay, mycelium samples (with three biological replicates of each sample) were carefully collected every 24 h, from day 10 to day 15 of culture, and stored at −80°C until use for RNA extraction. The qPCR was performed with a three-step method, using the real-time fluorescence quantitative PCR cycler *realplex2* (Eppendorf, Hamburg, Germany). Gene-specific primers were designed for qPCR assays (Supplementary Table S1). The *G. frondosa β-tubulin* gene was used as the internal reference. All qPCR reactions were determined for two independent experiments.

The relative expression levels were calculated, based on the 2^−ΔCt^ method, and analyzed by GraphPad Prism version 6.04 software.

### Vector construction and Pichia pastoris transformation

The codon sequence of the *hgfII* gene was optimized, according to the *P. pastoris* codon preferences, and synthesized by GENEWIZ (Suzhou, China). The target sequence of *hgfII* was amplified with the specific primer pair *hgfII* F and *hgfII* R (Supplementary Table S1), using the recombinant plasmid pUC57-*hgfII* as the template. The KEX2 and *Xho*I restriction sites were added to the upstream primer, while the downstream primer was designed with an *Eco*RI restriction site and a 6 × his tag. Both the PCR product and the plasmid pPIC9 were digested independently with *Xho*I and *Eco*RI (Takara, Beijing, China). Subsequently, the digested fragment products were ligated with pPIC9 and transformed into DH5α competent cells (Takara, Beijing, China). Positive transformants were selected by colony PCR and confirmed by sequencing (BGI, Beijing, China).

The corrected plasmid pPIC9-*hgfII* was linearized with *Stu*I at 37°C overnight, and was then transformed into GS115 competent cells according to the experimental methods described in our previous study (Niu et al., 2017).

### Protein expression and purification

In this study, batch-fed fermentation was carried out to induce the expression of HGFII as described in our previous studies (Song et al., 2016; Yang et al., 2019). A high yield yeast strain was selected for further expansion of the fermentation process. After 60 h of methanol-induced fermentation, the supernatant was collected by centrifugation, followed by a two-step purification: ultrafiltration and Ni-IDA His Bind Resin column (7 sea biotech, Shanghai, China) affinity purification. The target protein was identified by 12% SDS-PAGE gel and subsequent western blotting. The anti-His tag mouse monoclonal antibody-HRP was purchased from Shine Gene (E022330; Shanghai, China). HGFI-antibody was already available in our laboratory. Furthermore, matrix-assisted laser desorption/ionization time-of-flight (MALDI-TOF) mass spectrometry analysis was carried out to confirm the molecular weight of HGFII. Protein concentration was quantified using the BCA Protein Assay Kit (23225; Thermo Fisher Scientific, Shanghai, China).

### Water contact angle assay

Siliconized and untreated glass slides were used as the hydrophobic surfaces. The glass surfaces, with a uniform degree of silicification, were treated with 100 μl/ml HGFII aqueous solution at room temperature overnight. The treated surfaces were washed gently with deionized water the following day, then slowly dried with nitrogen gas. A HARKE Contact Angle Measurement goniometer (YH-168A; Beijing, China), using a 5 μl distilled water droplet, was used to measure the contact angle on HGFII-coated siliconized and untreated glass surfaces. A minimum of three independent measurements were performed with each treatment. The angle of each measurement was calculated using the Draw Tool software.

### X-ray photoelectron spectroscopy analysis

An aliquot of 100 μl/ml HGFII aqueous solution was incubated on a 1 × 1 cm siliconized glass slide at room temperature. X-ray photoelectron spectroscopy (XPS) was performed with a Thermo Fisher Scientific ESCALAB 250Xi instrument (21716057; Waltham, United States) to analyze the elemental composition of the untreated and HGFII-treated siliconized glass surfaces. The source gun type selected during the measurement was an A1 K Alpha, the lens mode was standard, pass energy was 100.0 eV, and the energy step size was set at 1.000 eV.

### Circular dichroism assay

Purified HGFII protein powder was dissolved in distilled water at a concentration of 50 μg/ml, then a 200-μl aliquot was added to the detection cell for circular dichroism (CD) measurement. The spectra were monitored using a MOS-450 spectrometer (Biologic, Claim, France) at a band width of 1 nm. The light source for the measurement was a 150 W xenon lamp, with the detection wavelength range from 190 to 250 nm. Scan internal was set at 1 nm.

The final records of each measurement was analyzed with the DichroWeb (Lighezan et al., 2013) program to predict the percentages of α-helix, β-strand, turn, and unordered region in the secondary structure of the HGFII protein solution.

### Thioflavin T fluorescence measurement

Thioflavin T (ThT) is a fluorescent dye that binds rapidly and specifically to the β-sheet fiber structure (Biancalana and Koide, 2010). It has an absorption peak at 485 nm. The interaction between ThT and HGFII solution before and after vortexing was detected with a Cary Eclipse Fluorescence Spectrophotometer (Agilent Technologies, Beijing, China). Solutions of 75 μg/ml HGFII and 5 mM ThT was prepared in distilled water, and 400 μl HGFII aqueous solution was transferred to the micro-injection pool along with 1.2 μl ThT for the assay. The spectrum was studied, with an excitation wavelength of 435 nm, whereas the scanning wavelength was set from 450 to 600 nm and the slit width was set to 10 nm. In this experiment, a ThT aqueous solution, in the absence of hydrophobin, was used as background.

### Atomic force microscope observation

In order to observe the self-assembled structure of HGFII in more detail, atomic force microscope (AFM) images were obtained with a Dimension Icon instrument (Bruker, Karlsruhe, Germany). Samples were prepared as described in a previous study (Yang et al., 2019). In brief, an aliquot (20 μl) of 100 μg/ml HGFII solution was pipetted onto a cleaned mica surface for 2 min, then the floating layer on the surface was carefully removed, washed with Milli-Q water three times and finally gently dried with nitrogen gas. All images were taken with a resolution of 512 samples/line, aspect ratio 1.0, and a scan rate ranging from 1 to 2 Hz. The recorded images were carried out with the NanoScope Analysis 1.5 software.

### Fluorescence intensity of 1-anilino-8-naphthalenesulfonate

Surface hydrophobicity is one of the most important features of hydrophobins. 1-Anilino-8-naphthalenesulfonate (1,8-ANS) is a fluorescent dye, which is commonly used to detect the surface hydrophobicity of proteins (Singh et al., 2019). In this experiment, the binding of 1,8-ANS to hydrophobins was studied with a Cary Eclipse Fluorescence Spectrophotometer (the same instrument as used to record ThT fluorescence) by setting the excitation wavelength at 370 nm. The emission spectra were recorded in a range from 390 to 700 nm. Slit widths were set at 10 nm for excitation and emission. An aliquot of 500 μl HGFII solution (100 μg/ml) was mixed with 1.25 μl 1,8-ANS (8 mM) for the assay. An aliquot of 500 μl 1,8-ANS (20 M) or 500 μl HGFII aqueous solution was used as control.

### Oil–water emulsification experiment

Hydrophobins are referred to as surfactants, because of their amphiphilic features. An emulsification experiment was carried out to identify the effect of HGFII on the dispersion of soybean oil in water. The tests were accomplished in glass vials. An aliquot of 2 ml (100 μg/ml) HGII aqueous solution was added to the vial, to which were added 120 μl soybean oil, followed by vortexing for 1 min, then sonication was conducted for 30 min. Subsequently, all the vials were placed on a flat surface at room temperature. Photos were taken after 24 and 72 h, respectively.

### Statistical analysis

Statistical analysis was performed using the GraphPad Prism version 6.04 software. Multiple *t*-tests (*p* ≤ 0.001) were performed to calculate the significance differences of the relative gene expression between *hgfII* and *hgfI*. Unpaired *t*-test (*p* < 0.05) was used to calculate the significance differences of the number droplets between HGFII and HGFI in the Oil–water emulsification experiment.

## Results

### Identification of *hgfII* in *Grifola frondosa*

**Figure 1:**
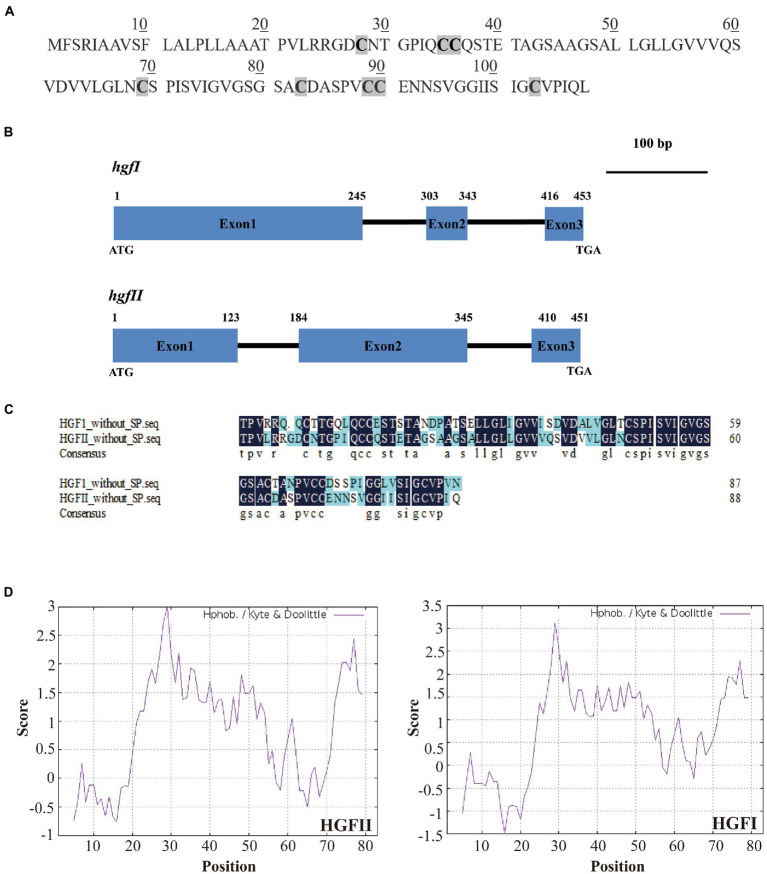
Identification and characterization of the novel hydrophobin gene *hgfII*. **(A)** The deduced protein sequence of HGFII. **(B)** Schematic diagram of genomic DNA sequences of *hgfII* and *hgfI*. **(C)** Sequence alignment between HGFII and HGFI without the signal peptides. **(D)** Hydropathy plot analysis of HGFII.

In the current study, we constructed a cDNA library from the mycelium which was harvested from a suspension culture of *G. frondosa* in PDB liquid medium. From this cDNA library, a new candidate hydrophobin gene was identified by colony PCR, using F1/R1 as primers (Supplementary Table S1). Subsequently, we named this gene *hgfII* (NCBI accession no. MW592703), according to the nomenclature rule of *hgfI* (NCBI accession no. EF486307). The length of *hgfII* cDNA was 327 bp and the corresponding protein sequence contains 108 amino acids which had a characteristic pattern of 8 Cys residues (Figure 1A). The full length of *hgfII* genomic DNA was 451, it contains two introns with lengths of 60 and 64 bp (Figure 1B). The predicted protein sequence of HGFII had a signal peptide at its N-terminus (Supplementary Table S2). Sequence alignment suggested a 61.08% identity between HGFII and HGFI without Signal peptide (Figure 1C).

Hydropathy plot analysis of the deduced protein sequence of HGFII showed that it shared a similar hydrophobicity stretch with HGFI (Figure 1D) which indicated that *hgfII* was a class I hydrophobin gene.

### Transcriptional level assay of *hgfII* and *hgfI* in mycelium

**Figure 2:**
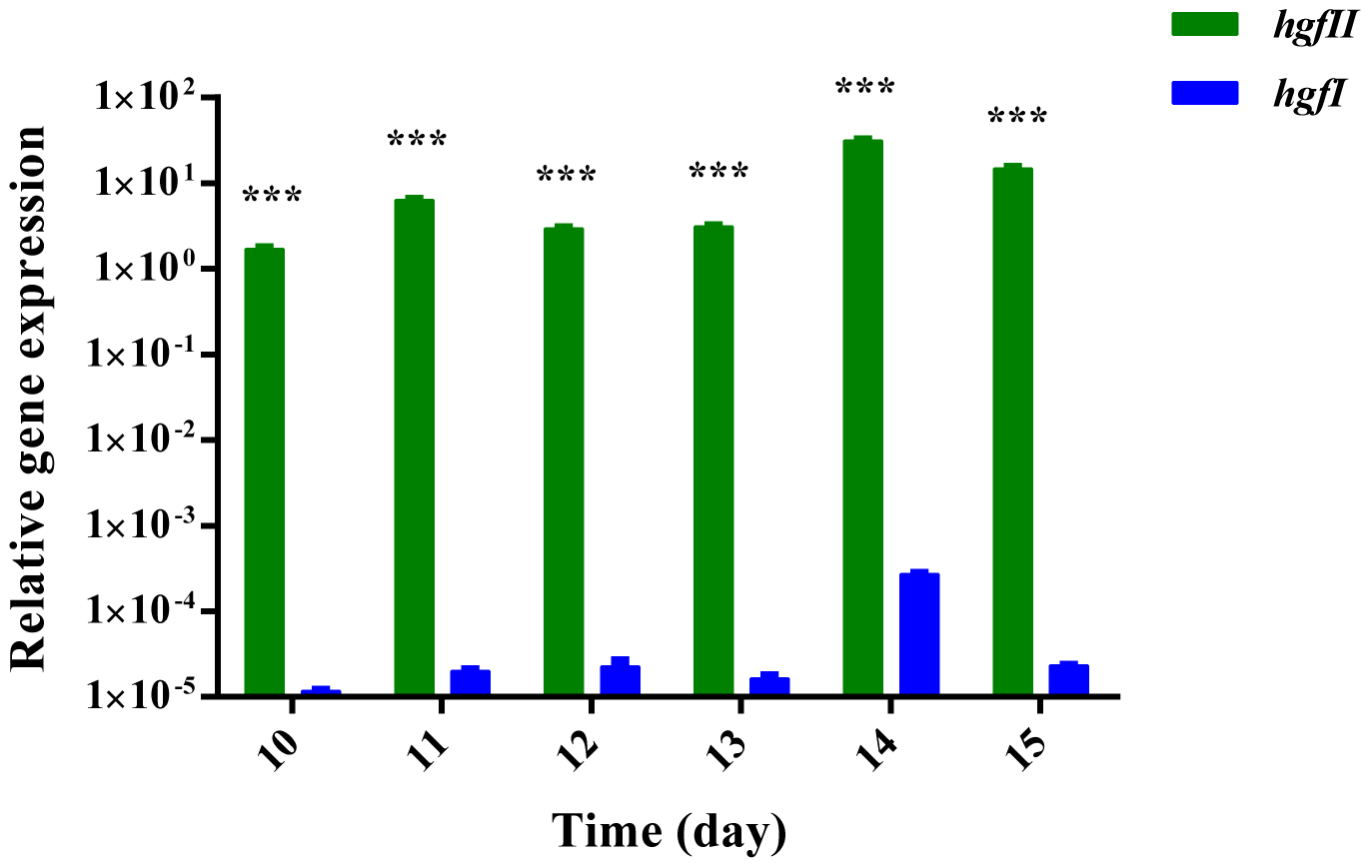
Relative gene expression of *hgfII* and *hgfI* in the hyphae of *Grifola frondosa* cultivated in liquid medium. Statistically significant difference: ^***^p ≤ 0.001 (Multiple *t-*tests).

To evaluate whether there is a difference between *hgfII* and *hgfI* at the transcriptional level, expression of the two genes was compared. At six time points, one per day, from day 10 to day 15, mycelium was harvested and the levels of mRNA were determined. As shown in Figure 2, there was a weak expression of *hgfI* in the mycelium harvested from the liquid medium. On the other hand, *hgfII* exhibited much stronger expression, which was almost 10^5^-fold higher than that of *hgfI*. These results suggested that *hgfII* may play a key role during the growth and development of hyphae cultured in liquid medium.

### Heterologous expression of *hgfII* in *Pichia pastoris* and protein purification

Based on the qPCR results, it was clear that *hgfII* exhibited a markedly higher transcription rate than *hgfI*. To further study the protein properties of HGFII, heterologous expression of HGFII with His-tag was carried out in *P. pastoris*. The open reading frame (ORF) of *hgfII*, without the coding sequence of the signal peptide, was optimized, according to the codon preferences of *P. pastoris*, and synthesized, to ultimately produce the recombinant plasmid pUC75-*hgfII*. The DNA fragment of *hgfII* amplified from pUC75-*hgfII* and the plasmid pPIC9 were digested with *Xho*I/*Eco*RI, respectively, and then used to generate the recombinant plasmid pPIC9-*hgfII*. The positive transformants, which showed a single 750-bp band, were selected for further verification. Subsequently, the recombinant plasmid, pPIC9-*hgfII*, was transformed into competent GS115 cells. The monoclone, which showed two bands (2 kb and 750 bp), using *AOXI* primers, as well as a specific band at 250 bp, using *hgfII* primers, was the positive transformant of *P. pastoris*.

**Figure 3:**
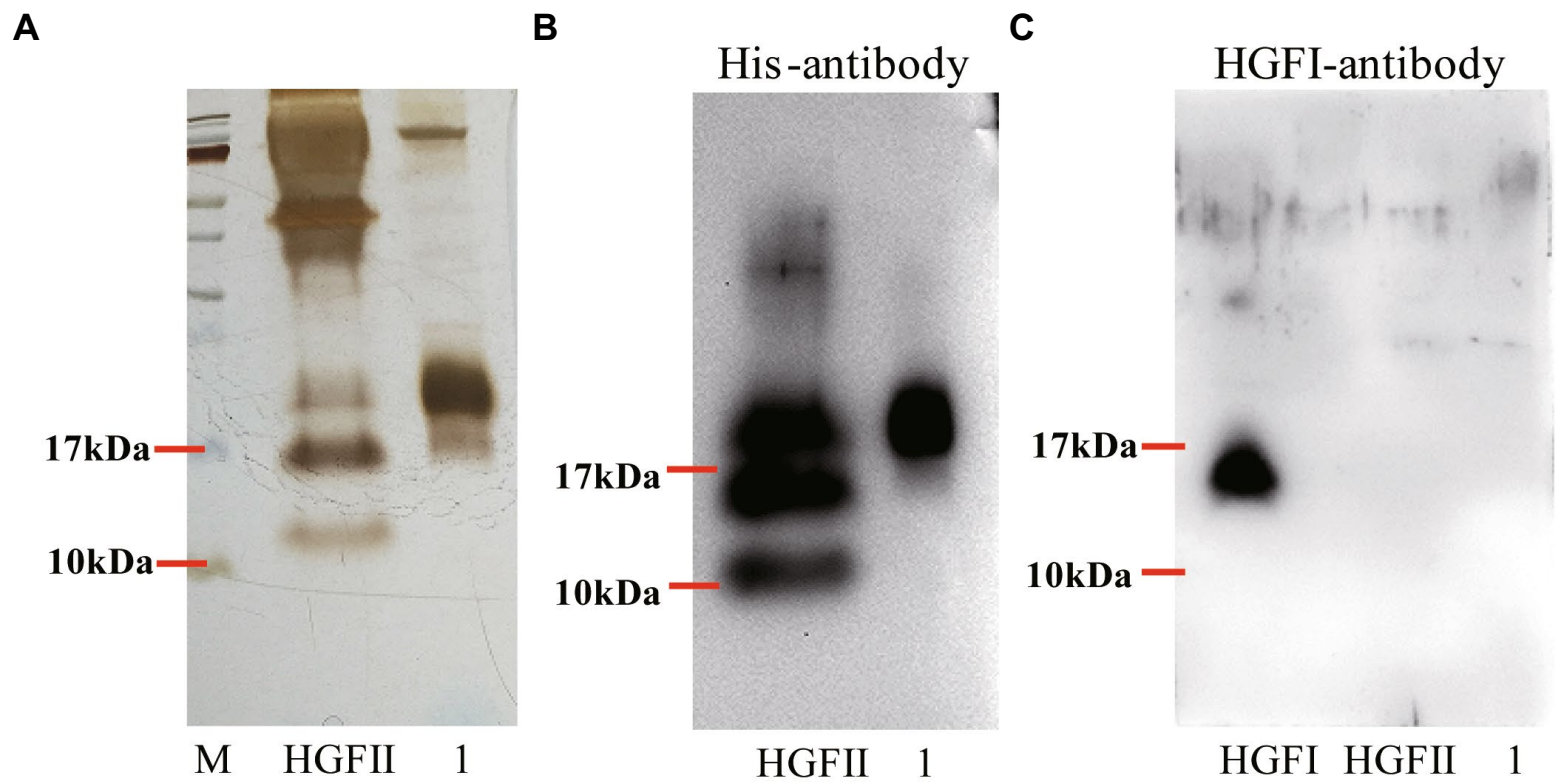
Purification of HGFII with His-tag in *P. pastoris*. **(A)** SDS-PAGE assay of HGFII. Western blot identification of HGFII with His-antibody **(B)**, and HGFI-antibody **(C)**. Lane “HGFII” is a crude extract of HGFII, lane 1 is pure HGFII after ultrafiltration and Ni-IDA purification.

The expression of HGFII was induced by methanol during the fermentation process. As shown in Figure 3A, the purification of HGFII, by Ni-IDA affinity purification and ultrafiltration, showed a single specific band on SDS-PAGE. This indicated that these purification methods were appropriate. Following this step, Western blotting was carried out using His-antibody (Figure 3B) and HGFI-antibody (Figure 3C). It was obvious that both the crude protein and the pure protein contained common specific bands in Figure 3B, results which were consistent with those from SDS-PAGE. However, in Figure 3C, we can see that no bands were apparent in either the HGFII crude protein or the corresponding purified protein. The molecular weight of the pure HGFII was also confirmed by MALDI-TOF (Supplementary Figure S1), the measured molecular weight corresponding to the predicted value.

### Water contact angle assay

**Figure 4:**
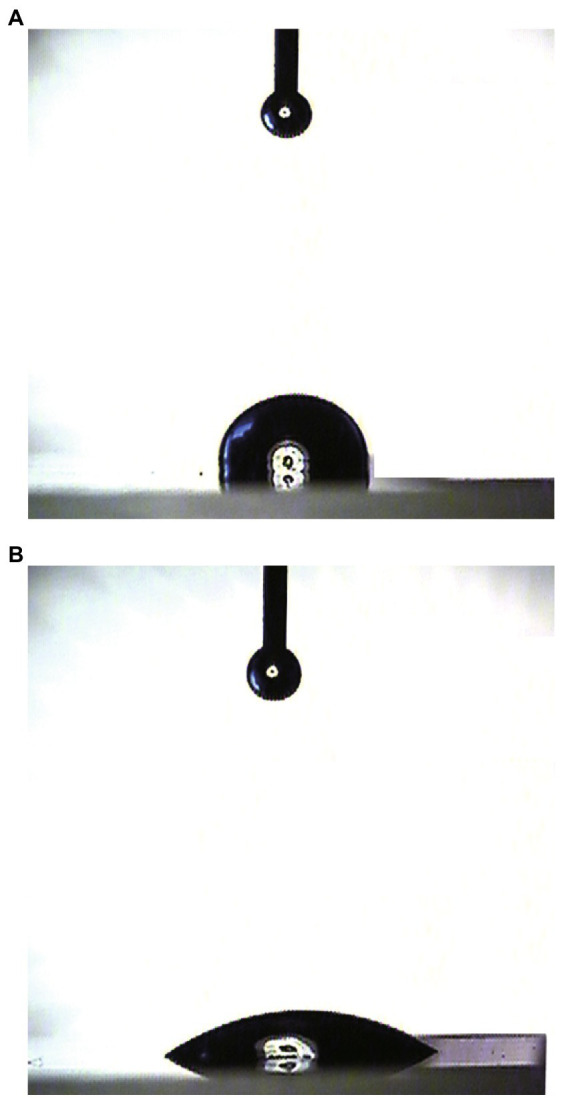
Water contact angle measurement of the surface modification ability of HGFII. **(A)** Uncoated siliconized glass. **(B)** HGFII-coated siliconized glass.

Surface modification ability is one of the most important properties for hydrophobins. Here, WCA was carried out to measure the wettability and hydrophobicity of both the HGFII-treated and -untreated siliconized glasses. As shown in Figure 4B, the water surface tension on HGFII-modified siliconized glass was much lower than that of the unmodified siliconized glass. Meanwhile, the WCA of the blank siliconized glass decreased from 87.6 to 32.5°C after treatment with HGFII (Table 1). This result demonstrated that HGFII had successfully self-assembled on the surface of the siliconized glass, thereby altering its surface hydropathy.

**Table 1:** The water contact angle of HGFII modified siliconized glass compared with HGFI.^a^

	Bare surface (deg)	HGFII-coated (deg)	HGFI-coated (deg)
Siliconized glass	87.6 ± 0.3	32.5 ± 3.5	54.1 ± 2

a The water contact angle of HGFI reference to our previous study (Yu et al., 2008).

### X-ray photoelectron spectroscopy analysis

**Figure 5:**
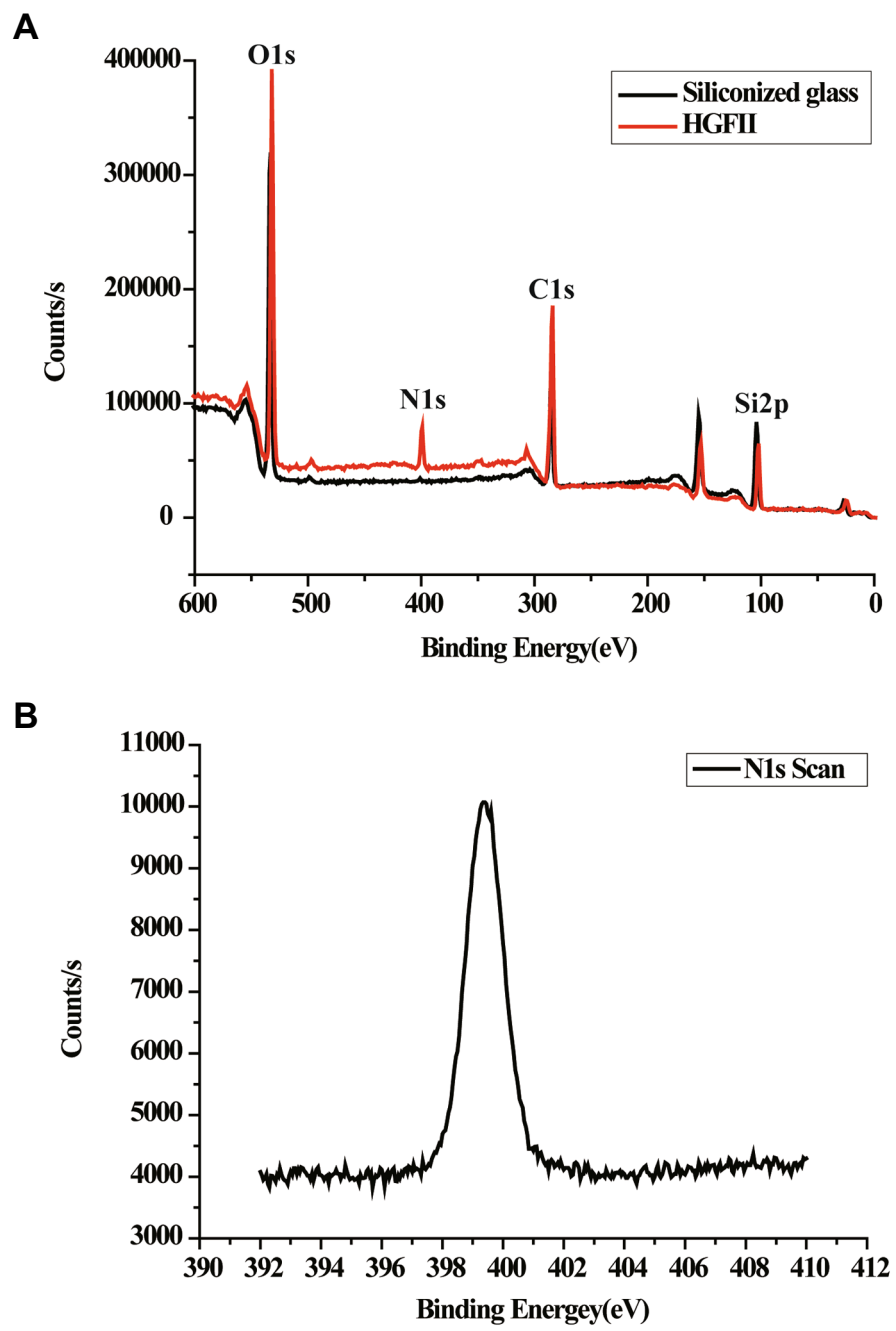
XPS survey of HGFII-coated siliconized glass. **(A)** Full-scan XPS survey of O1s, N1s, C1s, and Si2p. **(B)** N1s XPS curve of HGFII-coated siliconized glass.

In order to validate the WCA results, further characterization of the chemical element distribution of the HGFII-coated siliconized glass was performed. Results are shown in Figure 5. The survey scan was displayed, ranging from 0 to 600 eV. Figure 5A shows the variation in the contents of O, N, C, and Si. It was obvious that a new N peak appeared on the surface of the HGFII-treated glass surface, and its ratio increased from 0% to 6.08% on the modified glass (Figure 5B), whereas the content of Si decreased from 31.41% to 15.28%. These data revealed that the chemical nature, especially the elemental composition, of the HGFII-treated and untreated surfaces differed significantly.

### Circular dichroism assay

**Figure 6:**
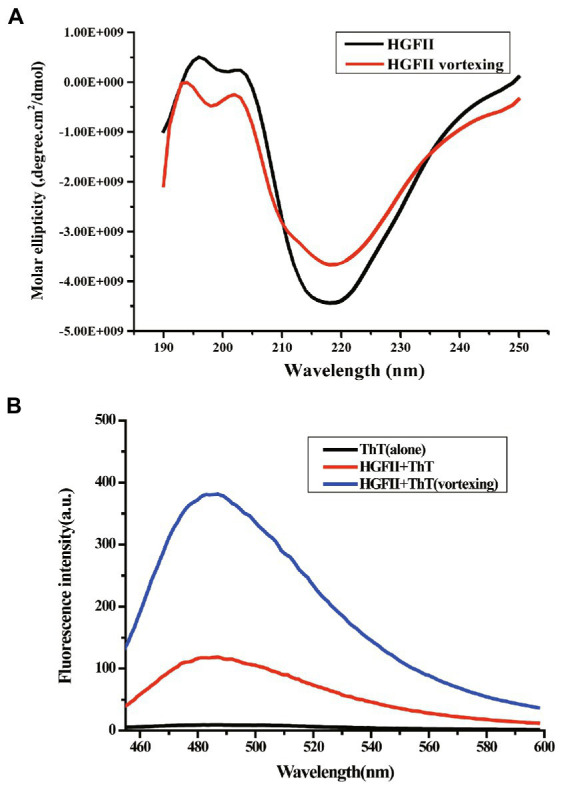
Characterization of protein self-assemble capacity. **(A)** Circular dichroism spectra of HGFII before (black curve) and after vortexing (red curve). **(B)** ThT fluorescence intensity assay of HGFII.

In the previous results, we hypothesized that HGFII is a member of the class I hydrophobin and it’s known to us that it undergoes a change of the secondary contents during the self-assembly process of class I hydrophobins. Thus, we further investigated the self-assemble property of HGFII on air–liquid surface using CD measurements. In Figure 6A, we can see that the aqueous solution of HGFII exhibited a strong negative peak at 218 nm under normal conditions (the black curve). By contrast, following vortexing of the aqueous solution (the red curve), HGFII showed a significant weakening of the negative peak. The secondary structure content of each spectrum was determined with the DichroWeb program, and results were summarized in Table 2. Similar to HGFI, the content of α-helix was greater than 70% in HGFII under normal conditions. Once vortexed, however, its α-helix content was reduced to 27.6%, with the percentage of β-sheet increasing from 12% to 40%. These results demonstrated that HGFII had successfully self-assembled at the air–liquid interface after vortexing, and we also inferred that HGFII may have the ability to self-assemble into rodlets.

**Table 2:** The contents of secondary structures of HGFII and HGFI.^b^

	α-Helix (%)	β-Strand and turn (%)	Unordered (%)
HGFII	74	12	14
HGFII vortexing	27.6	40	32.4
HGFI	77.4	7.1	15.4
HGFI vortexing	32.9	35	32

b The secondary structure values of HGFI reference to our previous study (Yang et al., 2019).

### Thioflavin T fluorescence measurement

To verify the speculation from the CD measurements, we carried out the ThT fluorescence assay. ThT is a benzothiazole dye which can be used as a fluorescence label because of its ability to bind with surfaces rich in β-sheets (Wang et al., 2017b). The ThT absorption peak reached about 100 a.u. as soon as 20 μM ThT was added into the HGFII aqueous solution without any treatment (the red curve; Figure 6B). After the reaction mixture was subjected to vortexing for 10 min, an enhancement of the fluorescence was observed (blue curve; Figure 6B), which may be caused by the abundant β-sheet aggregates in response to vortexing, along with the self-assembly of HGFII, thus leading to an increased interaction with ThT. These results supported the speculation that HGFII exhibits the ability to self-assemble into rodlet structures.

### Atomic force microscope observation

**Figure 7:**
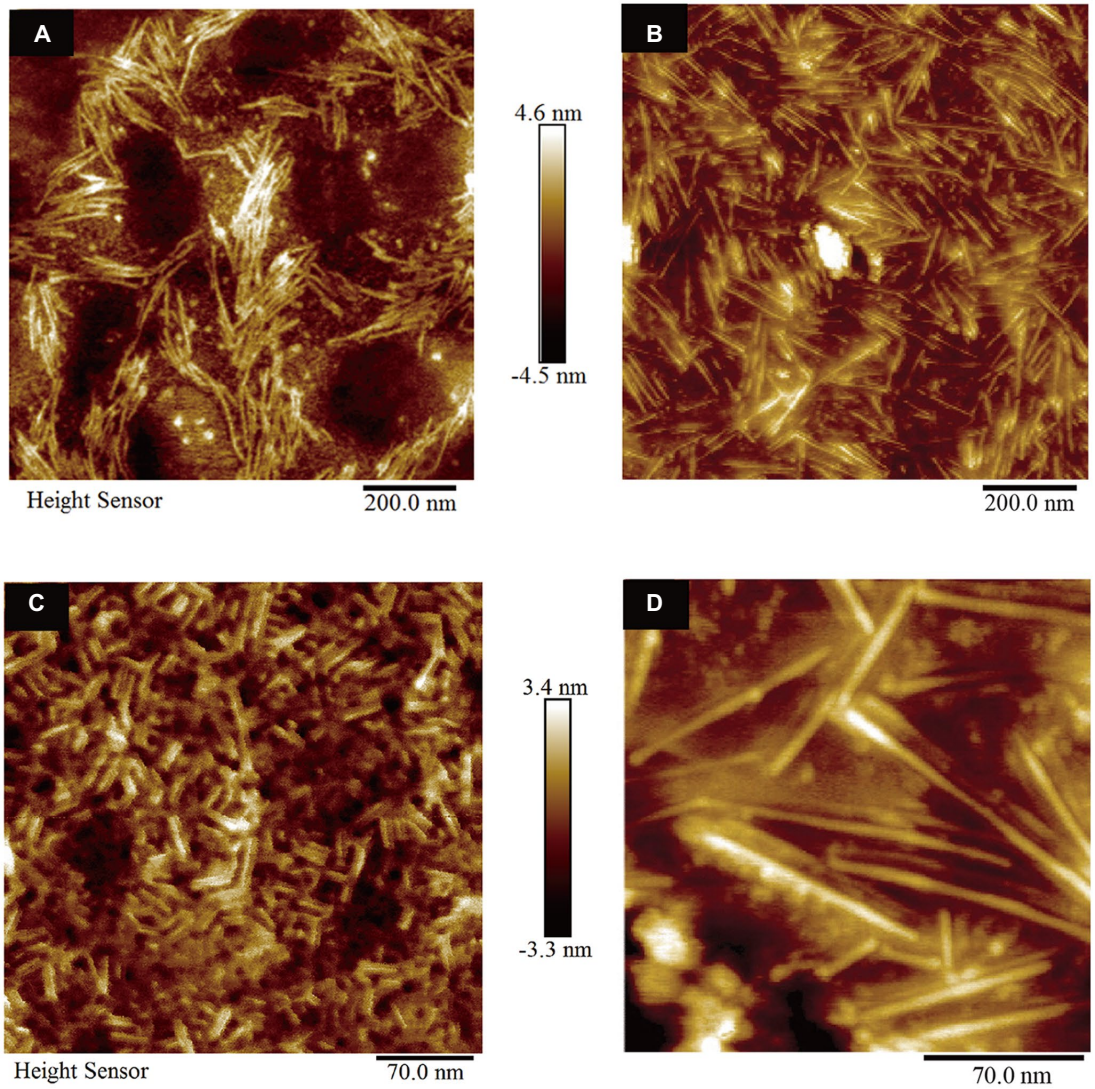
Surface morphology observation of hydrophobin-modified mica (*n* = 3). **(A)** AFM image of mica coated with HGFI-His. **(B)** AFM image of HGFI-coated mica. **(C)** AFM image of HGFII-coated mica. **(D)** AFM image of HGFI-coated mica. Images **(B,D)**, refer to our previous study (Yu et al., 2008).

According to the experimental data of CD spectra and ThT fluorescence assay, AFM measurements were carried out to observe the morphology of the self-assembled structures of HGFII. Before the measurement, we first analyzed whether the 6 × His-tag affected the self-assembled structure of the hydrophobins. The vector pPIC9-*hgfI*-His was constructed and transformed into GS115. Then, the protein HGFI-His, which contained a 6 × His at the C-terminus, was induced and purified by the same methods as used for HGFII. The surface topography of HGFI-His-modified mica was performed, using AFM. From the image shown in Figure 7A, we can see that HGFI-His can self-assemble into rodlet structures, of lengths in the range 100–150 nm, with no significant difference compared with the wild type HGFI (Figure 7B; Yu et al., 2008). This result indicated that the His-tag did not affect the structure of the self-assembled class I HGFI hydrophobin.

The morphology of HGFII was further investigated. As shown in Figure 7C, HGFII exhibited the ability to self-assemble into rodlets, with this result demonstrating that it was indeed a class I hydrophobin. On the other hand, the length of the rodlet-like structure formed by HGFII was between 15 and 30 nm, which was much more shorter than the HGFI rodlets (Figure 7D; Yu et al., 2008). This reflects the difference in rod formation ability between HGFI and HGFII.

### Fluorescence intensity of 1-anilino-8-naphthalenesulfonate

**Figure 8:**
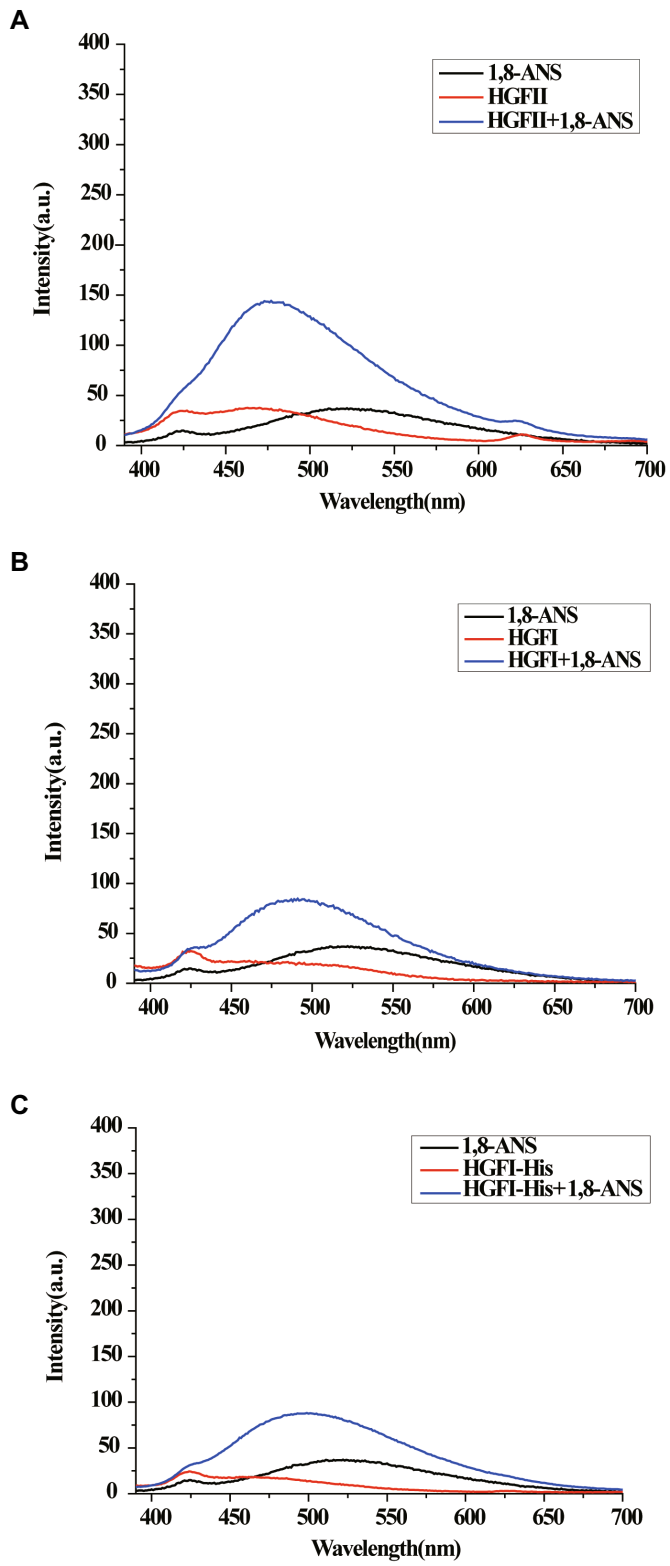
Surface hydrophobicity measurements. **(A)** The emission spectrum of 1,8-ANS in the presence of HGFII. **(B)** The emission spectrum of 1,8-ANS in the presence of HGFI. **(C)** The emission spectrum of 1,8-ANS in the presence of HGFI-His.

Here, 1,8-ANS was used as a fluorescent probe to characterize the hydrophobicity of HGFII. From Figure 8C we can see that the emission spectrum of 1,8-ANS in the presence of HGFI-His exhibited a slight increase at about ~490 nm, although no significant difference from the HGFI spectrum (Figure 8B). This suggested that the His-tag had no effect on the hydrophobicity of the hydrophobin. Interestingly, a 1.7-fold increase of the intensity was detected in the presence of HGFII (Figure 8A). Meanwhile, the maximum absorption value of HGFII was recorded at ~477 nm, exhibiting a distinct shift compared with HGFI (~490 nm). These results indicated that HGFII displayed a higher surface hydrophobicity than HGFI.

### Oil–water emulsification experiment

**Figure 9:**
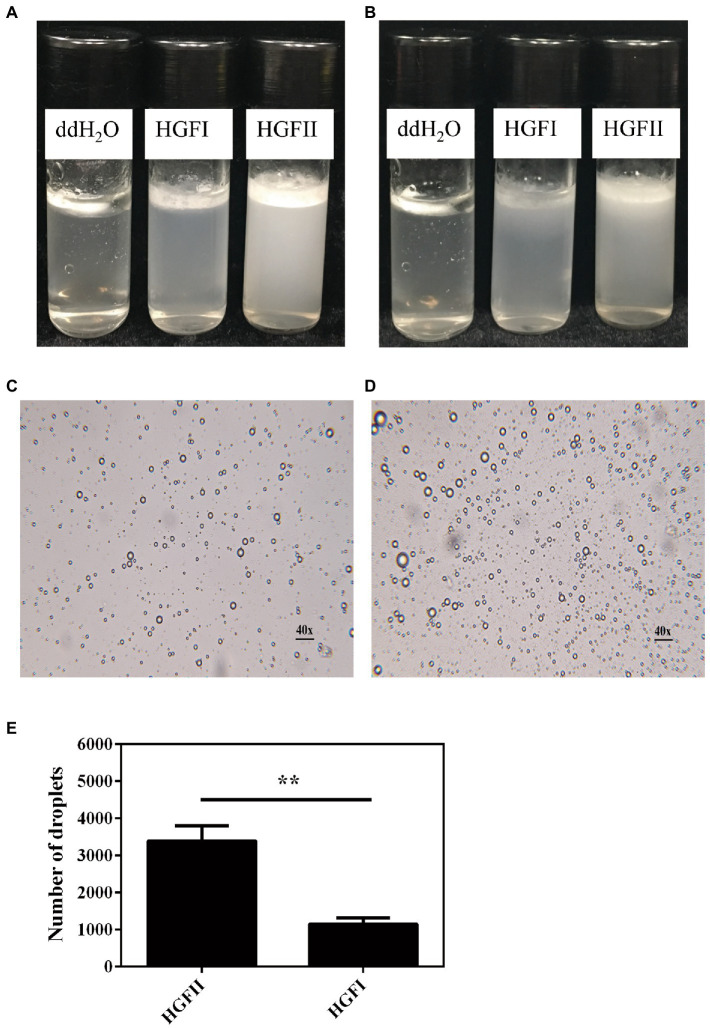
Emulsification of an oil–water mixed system after dispersion with HGFII and HGFI, respectively. Images were taken after the vials had been allowed to settle for 24 h **(A)** and 72 h **(B)** at room temperature. Microscope images of the oil droplets treated with HGFI **(C)** and HGFII **(D)** after standing for 24 h at room temperature. Scale label: 40◊ magnification. **(E)** Statistical analysis of the oil droplet numbers corresponding to image **(C,D)** with Image J and GraphPad Prism 6.04 programs [Statistically significant difference: ^**^p < 0.05 (Unpaired *t-*test)].

Finally, we tried to apply HGFII to the emulsification experiment, with HGFI being used as a control. As shown in Figure 9A, almost all the oil had accumulated in the upper layer of the vial at 24 h in the absence of hydrophobins, whereas the other two vials had formed emulsions, while HGFII displayed a better dispersive ability than HGFI. After extending the rest period to 72 h, visible delamination appeared in HGFI-dispersed emulsion. It’s obvious that the emulsification system dispersed by HGFII was more stable than HGFI (Figure 9B). The microscope images of the emulsion droplets at 24 h were observed under an optical microscope (Figures 9C,D). More emulsion droplets were formed by HGFII, almost 3.3-fold higher than HGFI-oil emulsion under the same field of view (Figure 9E).

## Discussion

Various fungi produce class I hydrophobins. As a result of their remarkable self-assembly characteristics, most of the research on hydrophobins has focused on their practical applications in different fields, so that the demand for different hydrophobins is increasing (Khalesi et al., 2015). So far, only one hydrophobin, HGFI, has been found in *G. frondosa* (Yu et al., 2008). In our present study, we recently found a novel hydrophobin gene *hgfII* from *G. frondosa*. The hydrophathy pattern of HGFII was similar with HGFI. In addition, the number of amino acids residues between the third and the fourth Cys residues of the deduced protein HGFII was 32, coinciding with a feature common to all class I hydrophobins (Ren et al., 2013). These data suggested that *hgfII* was a class I hydrophobin gene. Moreover, sequence alignment between the deduced protein sequence, without the signal peptide, of HGFII and HGFI revealed that they shared 61.8% identity which was similar to that between the hydrophobins SC3 and SC16 (Gandier et al., 2017). In this analysis, we can see that HGFII and HGFI were quite highly conserved. Thus, we concluded that HGFII belongs to the Class I *Basidiomycota* (Class IB) subdivision of hydrophobins.

As we know that HGFI was extracted from the mycelium harvested from PDA solid medium (Yu et al., 2008). In this study, we collected the hyphae of *G. frondosa* cultivated in PDB liquid medium and constructed a cDNA library. *HgfII* was identified from the library through PCR amplification. Analysis of the gene expression in liquid medium revealed that *hgfII* displayed a high transcriptional value at the late growth stage of mycelium. While the expression of *hgfI* was relatively too low to detect. This made it possible that *hgfII* may play a key role during the growth and development of liquid medium cultured hyphae.

Hydrophobins have been cited to be the most surface-active of all the known proteins (Wosten, 2001). They can spontaneously assembly into a protein film at hydrophobic/hydrophilic interfaces and thus altered interface to the opposite surface (Zhang et al., 2011; Ley et al., 2015). The WCA results of this study showed that the water contact angle of the bare siliconized glass decreased from a high hydrophobic value to a hydrophilic value (under 60°C) after co-incubated with HGFII. This indicated that HGFII had successfully self-assembled on the siliconized glass surface and associate altered it to a hydrophilic interface. On the other side, the XPS results reflected the formation of a protein membrane accompanied with the modification of HGFII on the hydrophobic interface. These results meant that HGFII possess the basic property of hydrophobins.

A remarkable characteristic during the self-assembly process of class I hydrophobin, was that the transition between the secondary structures consisting of the α-helix associated with the β-strand and turn (Wang et al., 2002; Gandier et al., 2017). Herein, the secondary structure contents of HGFII before and after vortexing treatment were performed with CD measurement. After vortexing for 10 min of the HGFII aqueous solution, the α-helix was reduced by 27.6%. While, the contents of β-strand and turn was increased up to 40% (Table 2). Interestingly, the fluorescence intensity of the binding assay between HGFII and ThT was significantly induced after the same treatment (Figure 6B). These results highlighted that HGFII holds the ability to self-assemble into rodlet structures. Further AFM observation had confirmed this hypothesis, we can see the rodlets clearly under a 350 nm microscope field with the diameter range from 15 to 30 nm (Figure 7C) which were significantly much shorter than that of HGFI. Hence, it was obvious that HGFII was indeed a class I hydrophobin and may has a distinct self-assembly capacity compared with that of HGFI.

Surface hydrophobicity is an important characteristic parameter of proteins, playing vital roles in not only maintaining the stability of protein but also affecting the protein function. Fluorescent probes are widely used to characterize the hydrophobicity of protein surfaces (Gasymov and Glasgow, 2007). Another aspect in which HGFII showed its difference with HGFI was reflected by ANS fluorescence assay. In our analysis, the interaction between HGFII and ANS was higher than HGFI, this indicated that the surface hydrophobicity of HGFII was stronger than HGFI.

In our experience, the self-assembly of HGFI at the oil–water interface was not particularly stable (Wang et al., 2017b). In this present study, soybean oil was used as a model to identify the self-assembly capacity of HGFII. As our results showed that HGFII exhibited a greater ability than HGFI to disperse the oil into aqueous solution. These proved HGFII to be more valuable as an emulsifier.

To conclude, in this present study, we isolated a novel hydrophobin gene, *hgfII*, from *G. frondosa* and successfully expressed it in *P. pastoris*. The very marked difference in the gene transcription rate between *hgfII* and *hgfI* indicated that *hgfII* may play a key role in the development of hyphae during suspension culture in liquid medium. A series of studies showed that the recombinant HGFII can self-assemble at the hydrophobic/hydrophilic interface. HGFII belongs to class I hydrophobin, it can self-assemble into a shorter rodlet structure which showed a significant difference with HGFI. In our analysis, the interaction with ANS was greater with HGFII than with HGFI, indicating that the surface hydrophobicity of HGFII was stronger than that of HGFI. Another aspect in which HGFII showed its difference from HGFI was shown in the emulsification assay, where HGFII displayed a better dispersion and emulsion stability in an aqueous solution with soybean oil than did HGFI. The above marked differences between HGFII and HGFI are of great significance in terms of expanding practical applications of class I hydrophobins, but also indicated that HGFII is a most interesting subject for future research.

## Data availability statement

The datasets presented in this study can be found in online repositories. The names of the repository/repositories and accession number(s) can be found in the article/Supplementary material.

## Author contributions

MQ, HX, and JY designed the experiments and wrote the manuscript. LG, BS, ZM, and XY performed the experiments. BW and YD analyzed the data. All authors contributed to the article and approved the submitted version.

## Funding

The research leading to these results was founded by the Sino-Swiss Science and Technology Cooperation project supported by the Ministry of Science and Technology of China (2015DFG32140). This work was also founded by the Youth Science Fund of the Natural Science Foundation of Tianjin (21JCQNJC00110) to JY. JY wants to acknowledge the financial support of the 2020 Tianjin Health Science and Technology Project, Science and Technology Talent Cultivation Project (KJ20070), and Chun Foundation of Tianjin First Central Hospital (2020CM12).

## Conflict of interest

The authors declare that the research was conducted in the absence of any commercial or financial relationships that could be construed as a potential conflict of interest.

## Publisher’s note

All claims expressed in this article are solely those of the authors and do not necessarily represent those of their affiliated organizations, or those of the publisher, the editors and the reviewers. Any product that may be evaluated in this article, or claim that may be made by its manufacturer, is not guaranteed or endorsed by the publisher.

## Footnotes

^1^ https://web.expasy.org/protscale/
