# Supplementary material for: A novel hydrophobin encoded by hgfII from Grifola frondosa exhibiting excellent self-assembly ability
Source: Front Microbiol. 2022 Sep 9;13:990231. doi: 10.3389/fmicb.2022.990231 (PMC9504065; doi:10.3389/fmicb.2022.990231)
Supplement: Supplementary file 1 [file Table_1.docx]

**Table S1 Primers used in this study**

| Primers | Primer sequence |
| --- | --- |
| F1 | 5'-TAATACGACTCACTATAGGG-3' |
| R1 | 5'-AGATGGTGCACGATGCACAG-3' |
| *hgfII* gF | 5'-ATGTTCTCTCGCATCGCTGCCGTTT-3' |
| *hgfII g*R | 5'-TCAGAGCTGGATTGGCACACAGCCA-3' |
| *β-tubulin* qF | 5'-AAGTTGGCTGTCAACATGGGT-3' |
| *β-tubulin* qR | 5'-GACGCAGCCATCATGTTCTTG-3' |
| *hgfI* qF | 5'-CAAGCTCGCCATCTTCGCTAC-3' |
| *hgfI* qR | 5'-GTGCGTCGACATCAGAGATG-3' |
| *hgfII* qR | 5'-GACGACGTCCACACTCTGG-3' |
| *hgfIII* qF | 5'-CCTCTCTGTTCTTGCGACCG-3' |
| *hgfII* F | 5'-CCGCTCGAGAAAAGAGGTGACTGTAACACCGGTC-3' |
| *hgfII* R | 5'-CGGAATTCTTAATGATGATGATGATGATGCAATTGGATTGGGACGC-3' |
| *AOXI* F | 5'-GCAAATGGCATTCTGACATCC-3' |
| *AOX1* R | 5'-GACTGGTTCCAATTGACAAGC-3' |
