# Supplementary material for: A novel hydrophobin encoded by hgfII from Grifola frondosa exhibiting excellent self-assembly ability
Source: Front Microbiol. 2022 Sep 9;13:990231. doi: 10.3389/fmicb.2022.990231 (PMC9504065; doi:10.3389/fmicb.2022.990231)
Supplement: Supplementary file 2 [file Table_2.docx]

**Table S2 The comparison of gene annotation between *hgfII* and *hgfI***

| **Gene name** | **cDNA**  **(bp)** | **Genomic DNA**  **(bp)** | **Translated protein**  **(aa)** | **Predicated signal peptide**  **(aa)** |
| --- | --- | --- | --- | --- |
| *hgfII* | 327 | 451 | 108 | 19 |
| *hgfI* | 324 | 453 | 107 | 19 |
